# Hypoglycemic and Hypolipidemic Effects of Triterpenoid Standardized Extract of *Agave durangensis* Gentry

**DOI:** 10.3390/plants14060894

**Published:** 2025-03-13

**Authors:** Juan David Bermudes-Contreras, Marcela Verónica Gutiérrez-Velázquez, Eli Amanda Delgado-Alvarado, René Torres-Ricario, Jorge Cornejo-Garrido

**Affiliations:** 1 Laboratorio de Biología Celular y Productos Naturales, Escuela Nacional de Medicina y Homeopatía, Instituto Politécnico Nacional (IPN), Gustavo A. Madero 07320, Ciudad de Mexico, Mexico; jbermudesc1800@alumno.ipn.mx; 2 Laboratorio de Biotecnología, Centro Interdisciplinario de Investigación para el Desarrollo Integral Regional (CIIDIR) Unidad Durango-IPN, Durango 34220, Durango, Mexico; mgutierrezv@ipn.mx (M.V.G.-V.); eadelgado@ipn.mx (E.A.D.-A.); rtorresr@ipn.mx (R.T.-R.)

**Keywords:** *Agave durangensis* Gentry, diabetes, triterpenes, DPP4, glycosylated hemoglobin, hypolipidemic

## Abstract

Diabetes mellitus is a chronic, degenerative, and multifactorial disease characterized by hyperglycemia, and at least 537 million people suffered from diabetes in 2021. *Agave durangensis* Gentry, a species of agave native to the state of Durango, reports phenolic compounds, flavonols, flavonoids, and saponins and could be an alternative for the treatment of diabetes. The aim of this work was to identify the compounds in the leaves of *Agave durangensis* Gentry and their potential activity in diabetes. The leaf extract of *Agave durangensis* Gentry (EAD) was characterized by ultra-performance liquid chromatography–mass spectrometry (UPLC-MS), and different families of bioactive compounds were quantified by analytical methods. Probable pharmacological targets were identified in silico, and the inhibition of dipeptidyl peptidase-4 (DPP4) was validated in vitro. A model of hyperglycemia was established with streptozotocin in male Wistar rats, and we administered EAD intragastrically at a dose of 300 mg/kg, as well as combinations of the extract with metformin and sitagliptin over 30 days. Biochemical and histological parameters were analyzed. We identified thirty-six major compounds, where triterpenes represented 30% of the extract. Molecular docking showed that the extract could interact with α-glucosidases and DPP4 since a large number of compounds in the extract have a Δ G lower than that reported for the controls, and DPP4 inhibition was confirmed by in vitro assays. In vivo assays demonstrated that the administration of the extract was able to significantly decrease glucose levels by 56.75% and glycosylated hemoglobin by 52.28%, which is higher than that reported for sitagliptin with a decrease of 35.22%. In addition, the extract decreased triglycerides by 59.28% and very-low-density lipoprotein (VLDL) cholesterol by 60.27%, and when administered in combination with metformin, it decreased them more than when metformin was administered alone. For all the above reasons, *Agave durangensis* Gentry extract could be used for the development of phytomedicine for the treatment of diabetes.

## 1. Introduction

Diabetes mellitus is a chronic degenerative and multifactorial disease characterized by elevated glucose levels (hyperglycemia) due to impaired insulin secretion, decreased insulin action, or both, where type 1 and 2 diabetes are the most common [1]. Type 1 diabetes is due to the destruction of β cells mediated by T cells and antibodies generated by B cells. This generates a depletion in insulin production. This is why this type of diabetes is considered insulin-dependent [2]. On the other hand, type 2 diabetes is a disease involving genetic and environmental factors, characterized by insulin resistance in muscle and liver and deficient insulin secretion by pancreatic β cells. These defects lead to excessive glucose production by the liver and in adipocytes produce accelerated lipolysis and increased plasma free fatty acid levels, which aggravate insulin resistance [3]. According to the International Diabetes Federation (IDF), at least 537 million people were suffering from diabetes in 2021, a number expected to reach 738 million by 2045. In addition, around 541 million people have glucose intolerance and at least 6.7 million people between the ages of 20 and 79 will die from diabetes-related causes [4]. Of this number of patients, 90–95% are expected to have diabetes mellitus type 2 (DM2), which can be treated with diet, exercise, and medication. Among these, the main families of oral medications include biguanides, where metformin is one of the first-line medications for the treatment of DM2; we also have sulfonylureas such as glibenclamide, glimepiride, etc. There are also thiazolinediones such as pioglitazone; α-glucosidase inhibitors such as acarbose and miglitol; DPP4 inhibitors such as sitagliptin, saxagliptin, etc.; and Sodium-glucose cotransporter-2 (SGLT2) inhibitors such as empagliflozin, dapagliflozin, etc. Finally, there is semaglutide, the most current oral drug of Glucagon-like peptide-1 (GLP-1) analogues [3,5,6]. Of these drugs, DPP4 inhibitors, SGLT2 inhibitors, and GLP-1 analogs have shown a better effect in lowering glucose levels, in addition to having fewer side effects than others; however, the cost of these is higher so it becomes a limiting factor for access to them and an impediment to achieve the goals in glycemic control. In view of this situation, research and development of new drugs has accelerated, with plants being an interesting alternative due to their medicinal properties, easy access, and lower costs than some of the drugs on the market.

Since ancient times, plants have been used to treat various diseases and have been found to contain bioactive compounds that can be used as a source of new medicines. In the pharmaceutical industry, up to approximately 80% of the compounds are derived from natural compounds or are developed from them [7]. In Mexico, more than 300 species of 235 genera and 93 families with hypoglycemic effects have been reported; however, it is estimated that this number is greater than 800 species. It has been found that the main families of bioactive compounds present in these plants are flavonoids, steroids, terpenoids, and oligosaccharides, among others [8,9]. The agave genus is one of the most important in Mexico because more than 80% of the world’s species are found here and more than 40% are endemic [10,11,12]. Agaves are plants that are utilized in the production of alcoholic beverages and fibers. In traditional medicine, Agave species have been used to treat wounds, sores, trauma, fractures, rheumatoid arthritis, psoriasis, snake bites, syphilis, scurvy, cancer, limb paralysis, and postpartum abdominal inflammation, as well as being used as diuretics and laxatives [13,14,15]. However, they have demonstrated the potential to possess intriguing pharmacological properties, including antidiabetic, anti-inflammatory, anticancer, antimicrobial, and antiulcerogenic effects. Additionally, they produce bioactive compounds of interest, such as phenolic compounds, saponins, and phytosterols [12,16,17].

One noteworthy species of agave is the *Agave durangensis* Gentry, which is one of the 29 documented species of agave in the state of Durango, Mexico. It is distributed in the municipalities of Nombre de Dios, Durango, and El Mezquital, as well as in the northern part of Zacatecas in the region that borders the municipality of El Mezquital. This plant is a succulent with spiral-shaped leaves that form a rosette. It has an average height of 80–120 cm and a width of 120–180 cm. Its leaves are gray-green, measuring 40–90 cm long and 14–22 cm wide. They are rough, waxy, with prominent teeth 1 to 2 cm in length, flattened, and spaced 1 to 2 cm apart, with a hard spine 4 to 6 cm long, gray-brown in color. The inflorescences are a panicle 7 to 8 m tall with approximately 18 to 30 upper branches. The flowers are upright and yellow, measuring 6–8 cm long. The seeds are small and oval-shaped [18,19] This species is of particular importance to the state due to its utilization in the production of mezcal. Two major agro-industrial wastes are generated during this process: bagasse and leaves, of which the leaves represent between 45 and 50% of the total weight of the plant [20]. It has been discovered that flowers contain a variety of compounds such as kaempferol-3,7-O-diglucoside, kaempferol-3-O-[rhamnosyl-(1-6)-glucoside], and quercetin-3-O-[rhamnosyl-(1-6)-galactoside] [21,22], in addition to having antioxidant activity [22]. On the other hand, the leaves have been shown to contain bioactive compounds such as kaempferol 3-O-glucuronide, kaempferol rhamnoside, kaempferol 3-O-coumaroyl glycoside, and other flavonoids [23] and saponins like Agavoside A and Agavoside B, in addition to exhibiting antibacterial and antifungal activity [24].

The aim of this work was to identify the compounds in the leaves of *Agave durangensis* Gentry and their potential activity in diabetes, and we found that the extract of this agave is rich in triterpenes; in addition to this, it was found that it is capable of inhibiting DPP4, demonstrating a decrease in glucose and lipid levels.

## 2. Results

### 2.1. Characterization of Agave durangensis Extract

The percentage extraction yield of *Agave durangensis* Gentry was 2.17%, which means that 21.7g was obtained per kilogram of plant material. In the EAD, the concentration of triterpenes was 294.30 ± 0.88 mg equivalents of ursolic acid per g (UAE/g) of extract, and the concentration of phenols was 0.019 ± 0.000 mg equivalents of gallic acid per g (GAE/g) of extract. Additionally, the concentration of flavonoids was 0.004 ± 0.000 mg equivalents of quercetin per g (QE/g) of extract, and the concentration of condensed tannins was 0.003 ± 0.000 mg equivalents of epicatechin per g (EE/g) of extract.

**Figure 1:**
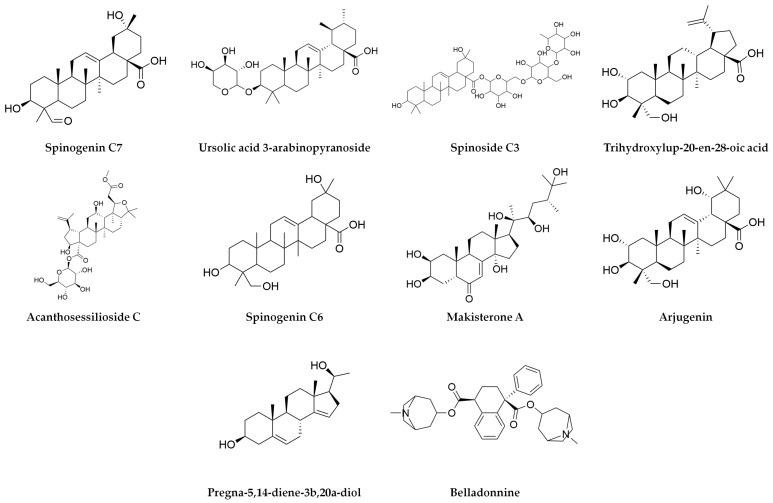
Compounds of *Agave durangensis* extract identified by UPLC-MS ESI in positive mode.

**Figure 2:**
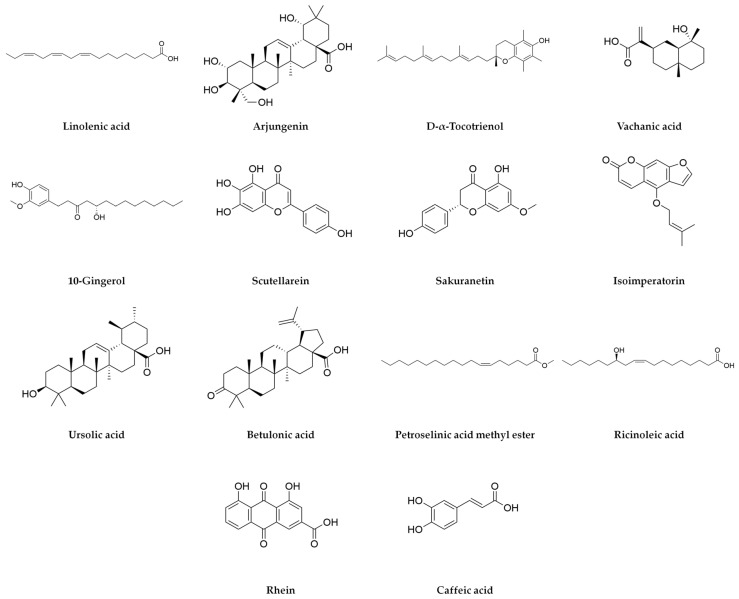
Compounds of *Agave durangensis* extract identified by UPLC-MS ESI in negative mode (part one).

**Figure 3:**
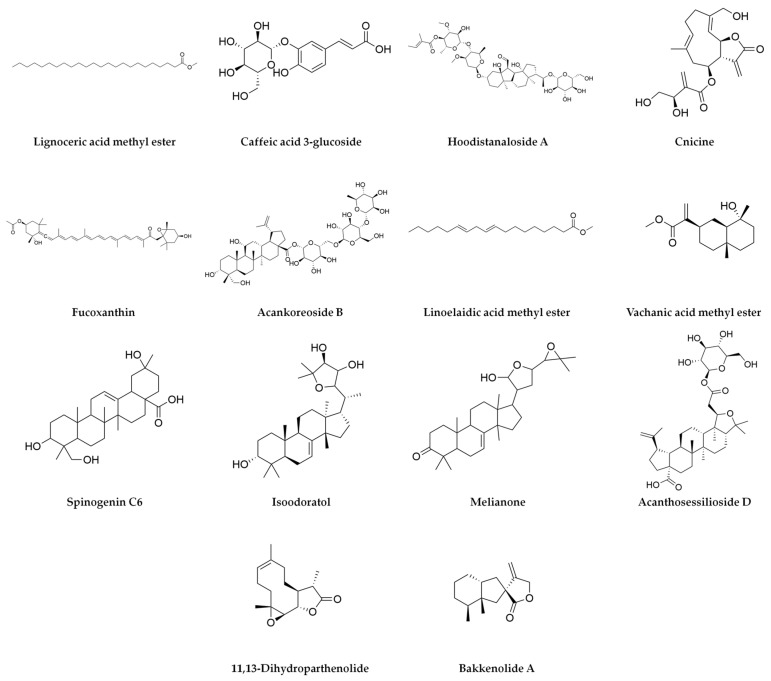
Compounds of *Agave durangensis* extract identified by UPLC-MS ESI in negative mode (part two).

A total of 36 bioactive compounds were identified in the *Agave durangensis* extract, as shown in Figure 1, Figure 2 and Figure 3. Comprehensive data for each compound including compound name, observed neutral mass, mass error, retention time, response, and observed fragments are presented in Table 1 and Table 2.

The ten major compounds of the EAD, as determined by the area under the curve, are linolenic acid, spinogenin C7, arjungenin, D-α-tocotrienol, vachanic acid, 10-gingerol, scutellarein, sakuranetin, isoimperatoin and ursolic acid, where triterpenes represent 30% of the extract.

### 2.2. In Silico Study of Agave durangensis Extract Bioactive Compounds

Molecular docking was performed with different drug targets, and all the compounds were identified by UPLC-MS. Table 3 illustrates the binding affinities (kcal/mol) of the different ligands, as well as those of the controls. The compounds with a delta G value lower than that reported for the control of each target are indicated in bold, indicating that they have a higher binding affinity.

In molecular docking, a more negative binding value is indicative of better binding. It can be observed that none of the bioactive compounds under investigation demonstrated a superior capacity to bind AMP-activated protein kinase (AMPK) and GLP1-R in comparison to the respective controls. On the other hand, Supplementary Table S1 shows the compounds that bind to different targets as well as the interactions present between these compounds and the proteins. It is important to note that for the most part, similar interactions were preserved between the bioactive compounds of the EAD and the controls, with amino acids reported as important for receptor activation or inhibition. It can be seen that only two bioactive compounds (fucoxanthin and D-α-Tocotrienol) had a lower binding energy than the control with SGLT2, and it was observed that six bioactive compounds (ursolic acid 3-arabinopyranoside, spinoside C3, hoodistanaloside A, acankoreoside B, belladonnine, melianone) bound to sulfonylurea receptor−1 (SUR1), 24 bioactive compounds (spinogenin C7, arjungenin, D-α-tocotrienol, scutellarein, sakuranetin, isoimperatorin, ursolic acid, betulonic acid, ursolic acid 3-arabinopyranoside, rhein, spinoside C3, trihydroxylup-20-en-28-oic acid, acanthosessilioside C, makisterone A, pregna-5,14-diene-3b,20a-diol, caffeic acid 3-glucoside, hoodistanaloside A, fucoxanthin, acankoreoside B, belladonnine, spinogenin C6, isoodoratol, melianone, acanthosessilioside D) bound to maltose-glucoamylase at its C end terminal, and 25 bioactive compounds (spinogenin C7, arjungenin, D-α-tocotrienol, scutellarein, sakuranetin, isoimperatorin, ursolic acid, betulonic acid, ursolic acid 3-arabinopyranoside, rhein, spinoside C3, trihydroxylup-20-en-28-oic acid, acanthosessilioside C, makisterone A, pregna -5,14-diene-3b,20a-d iol, caffeic acid 3-glucoside, hoodistanaloside A, cnicine, fucoxanthin, acankoreoside B, belladonnine, spinogenin C6, isoodoratol, melianone, acanthosessilioside D) at its N terminus. A total of 21 bioactive compounds (arjungenin, D-α-tocotrienol, vachanic acid, scutellarein, sakuranetin, isoimperatorin, ursolic acid, betulonic acid, ursolic acid 3-arabinopyranoside, rhein, spinoside C3, trihydroxylup-20-en-28-oic acid, acanthosessilioside C, hoodistanaloside A, fucoxanthin, acankoreoside B, belladonnine, spinogenin C6, isoodoratol, melianone, acanthosessilioside D) bound to sucrose-isomaltase and 23 bioactive compounds (spinogenin C7, arjungenin, D-α-tocotrienol, scutellarein, sakuranetin, isoimperatorin, ursolic acid, betulonic acid, ursolic acid 3-arabinopyranoside, rhein, spinoside C3, trihydroxylup-20-en-28-oic acid, acanthosessilioside C, makisterone A, pregna-5,14-diene-3b,20a-diol, hoodistanaloside A, fucoxanthin, acankoreoside B, belladonninel, spinogenin C6, isoodoratol, melianone, acanthosessilioside D) bound to pancreatic α-amylase, observing that 22 bioactive compounds are capable of binding to the entire set of enzymes called α-glucosidases. On the other hand, nine bioactive compounds (sakuranetin, ursolic acid 3-arabinopyranoside, spinoside C3, makistero ne A, hoodistanaloside A, acankoreoside B, belladonnine, isoodoratol, acanthosessilioside D) bound to peroxisome-proliferator-activated receptor gamma (PPARγ), and finally, 13 bioactive compounds (arjungenin, sakuranetin, ursolic acid, ursolic acid 3-arabinopyranoside, rhein, spinoside C3, hoodistanaloside A, fucoxanthin, acankoreoside B, belladonnine, isoodoratol, melianone acanthosessilioside D) bound to DPP4.

The pharmacokinetic properties of the compounds present in the EAD are presented in Supplementary Table S2. First, the physicochemical properties of the compounds are described, among which are the molecular weight (MW), the topological polar surface area (TPSA), the number of hydrogen bond acceptors (nHA) and donors (nHD), etc. The absorption parameter is also described in terms of the prediction of permeability in Caco-2 cells, in Madin–Darby canine kidney (MDCK) cells, as a substrate or inhibitor of P-glycoprotein, and in terms of human gastrointestinal absorption and human oral bioavailability. On the other hand, the distribution parameters, including blood–brain barrier crossing (BBB), plasma protein binding (PPB), volume of distribution (VD), and fraction unbound to plasma (Fu), are also predicted. The prediction of metabolism was carried out through the binding or inhibition parameters to different cytochromes including CYP1A2, CYP2C19, CYP2C9, CYP2D6, and CYP3A4. The excretion parameter was predicted through renal clearance (CL) and half-life time (T1/2). Finally, the toxicology heading includes the parameters of hepatotoxicity, carcinogenicity, mutagenicity, cytotoxicity, hERG channel blockade, and lethal dose 50 (LD_50_).

It was observed that the majority of the compounds under consideration exhibited characteristics that would allow them to be developed into drugs. These include good absorption, protein binding, and half-life. Furthermore, the majority of the compounds do not present toxicological effects.

### 2.3. DPP4 Inhibitory Activity In Vitro of Agave durangensis Extract

The in vitro enzyme inhibition study of DPP4 reveals that EAD is able to inhibit this enzyme since the concentrations tested that an IC_50_ of 861 µg/mL ± 129.8 µg/mL could be calculated, which reinforces what was suggested by the in silico studies.

### 2.4. Hypoglycemic and Hypolipidemic Effect of Agave durangensis Extract

**Figure 4:**
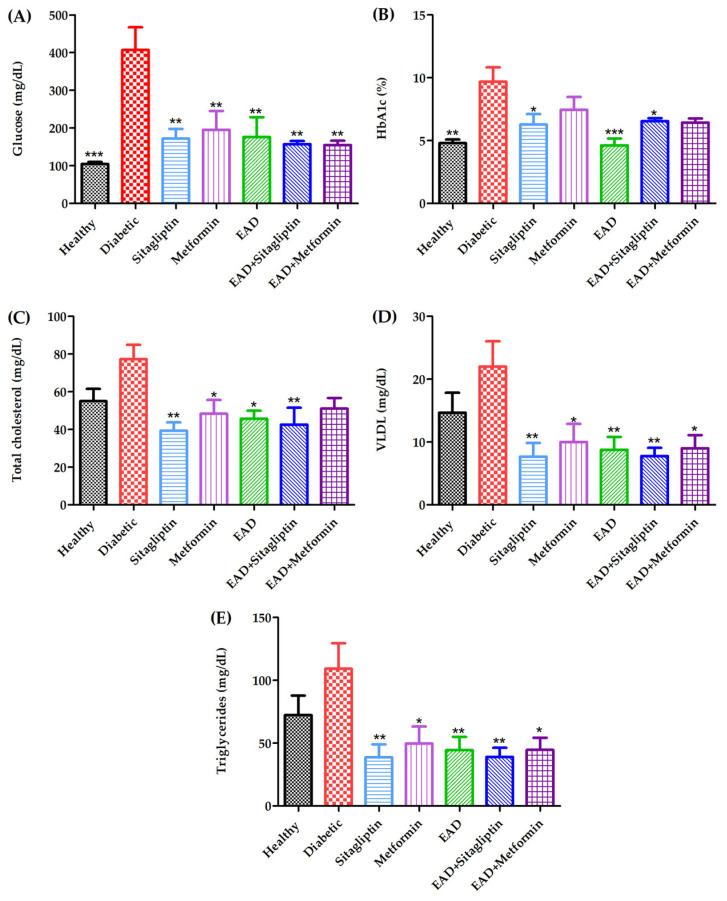
Effect of the administration of EAD and combinations in an animal model. (**A**) Glucose, (**B**) HbA1c, (**C**) total cholesterol, (**D**) VLDL, and (**E**) triglycerides levels. * *p* < 0.05; ** *p* < 0.01; *** *p* < 0.001 compared to the diabetic group.

The in vivo results demonstrate that EAD administration results in a reduction in glucose levels (Figure 4A), with 56.75% observed in comparison to the diabetic group. Furthermore, the levels attained are lower than those reported for reference drugs. Figure 4B shows the glycated hemoglobin (HbA1c) levels, which demonstrate a notable decline, in the groups treated with the extract, sitagliptin, and the combination of both. The EAD administration resulted in a 52.28% reduction in glycated hemoglobin levels in comparison to the diabetic group. In addition, a notable reduction in total cholesterol levels was observed across all the treated groups with the exception of the combination group of metformin with EAD (Figure 4C); this group exhibited a 40.88% decrease in cholesterol levels in comparison to the diabetic group. Figure 4D illustrates a notable decline in VLDL cholesterol levels across all treated groups, with the greatest reduction observed in the EAD, sitagliptin, and combined treatment groups. The administration of EAD alone resulted in a 60.27% reduction in VLDL cholesterol levels compared to the diabetic control group. A reduction in triglycerides levels was also observed in all treated groups (Figure 4E). This was most pronounced in the EAD, sitagliptin, and combination groups, where the administration of EAD decreased triglyceride levels by 59.28% compared to the diabetic group. On the other hand, it was decided to evaluate the combination of the extract with approved drugs used for the treatment of diabetes since most of the plant extracts can interfere with these drugs, decreasing their efficacy, so the joint evaluation allows for the ensuring that the effect is preserved or increased. From this, we observed that the combination of EAD with sitagliptin seems to have the same effect on the different parameters as when sitagliptin is administered individually. However, the combination group of EAD with metformin showed a more substantial decrease in glucose levels, with a decrease of 61.99% compared to 52.08% in the group that received metformin alone and in the levels of glycosylated hemoglobin where the combination decreased by 33.56%, while when metformin was administered alone, it only decreased by 23.06%.

In Table 4, remaining biochemical parameters can be observed, where we can see an increase in the levels of aspartate amine transferase (AST) and alanine amine transferase (ALT) without presenting a significant difference. It is noteworthy that there was a considerable elevation in urea levels observed in the diabetic groups and those treated with sitagliptin and metformin. Conversely, the groups that were administered EAD and the combinations did not show this significant increase. Regarding creatinine levels, an increase was observed in the diabetic group without being significant.

Table 5 shows the % of DPP4 activity in plasma, where the extract showed an activity of 74.72%, which represents a significant difference with respect to the diabetic group, suggesting that the extract does inhibit this enzyme in vivo, although not as efficiently as sitagliptin does, since the activity of DPP4 when this drug was administered was only 7.13%; on the other hand, when metformin was administered, the activity of the enzyme also increased. Finally, it was observed that the combination of extract with sitagliptin also decreased the activity of DPP4, being lower than when only sitagliptin was administered, and for the case of extract with metformin, we can observe 94.86% activity, which would indicate that the extract does inhibit DPP4, even in the presence of drugs.

**Figure 5:**
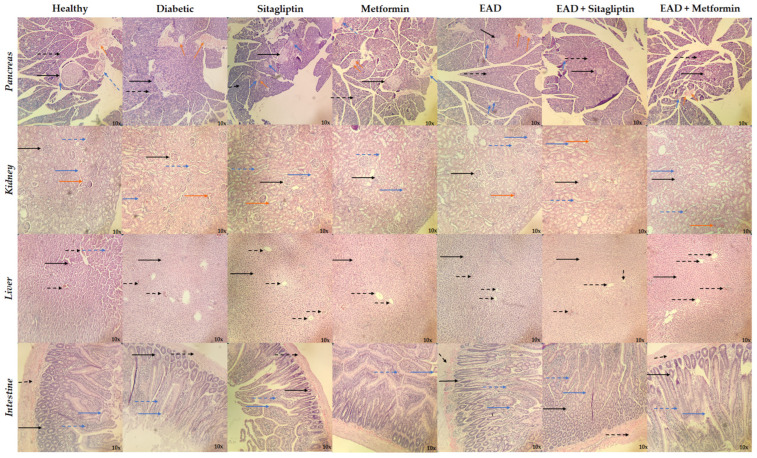
Histopathological analyses. The sections were stained with hematoxylin and eosin (H&E) and observed with a 10× objective. Pancreas: Islets of Langerhans (black continuous arrow), pancreatic acinus (dashed black arrow), intralobular duct (blue continuous arrow), interlobular duct (dashed blue arrow), blood vessel (orange continuous arrow). Kidney: glomerulus (black continuous arrow), proximal convoluted tubule (blue continuous arrow), distal convoluted tubule (dashed blue arrow), blood vessel (orange continuous arrow). Liver: hepatocyte (black continuous arrow), muscle (dashed black arrow). Intestine: crypt (black continuous arrow), pancreatic acinus (dashed black arrow), intestinal villi (blue continuous arrow), submucosa (dashed blue arrow).

As shown in Figure 5, the decrease in size, the change in shape, and the data on necrosis of the pancreatic islet were observed in all groups induced to hyperglycemia (diabetic, sitagliptin, metformin, EAD, EAD + sitagliptin, EAD + metformin), which is expected for the model used, while the healthy group retained the expected morphology for the pancreatic islet. On the other hand, no alterations in renal morphology were observed in any of the groups. Only the diabetic group presented data suggestive of liver damage, which is related to elevated levels of liver enzymes. Finally, there were no changes in the morphology of the intestine in any of the experimental groups.

## 3. Discussion

*Agave durangensis* Gentry is a species of agave that has been the subject of relatively limited scientific investigation, particularly in comparison to other species. Regarding the method of obtaining the extract, this is the first time that the use of ethyl acetate as a solvent is reported for this species of agave. There is no report of the percentage of extraction with any solvent for *Agave durangensis*, but there are reports for other agaves and other solvents. It has been reported that other solvents such as 80% methanol can increase extraction yields—for example, yields for *Agave americana* range from 9.83% when using fresh leaves [25] to 15.1% when using dried leaves [26]. On the other hand, low extraction percentages have been reported when using aqueous extracts—for example, *Agave angustifolia* reports a percentage of 0.57% and *Agave cupreata*, 0.93% [27]. Therefore, the yield may vary due to different conditions such as the use of fresh or dry material, the polarity of the solvent, or even the use of methods such as ultrasound or temperature. In addition, the increase in the extraction percentage may be due to the type of metabolites we are obtaining, where the extracts obtained with methanol will have a greater amount of high-polarity compounds while the extracts obtained with ethyl acetate may carry high- or low-polarity compounds.

Studies focused on knowing the phytochemical composition of *Agave durangensis* have focused solely on knowing the phenolic profile of the leaves, leaving aside the other families of compounds present. It is interesting to observe that the molecules found are different from those that had been reported in previous works where compounds such as kaempferol and derivatives, 3,4-dicaffeoylquinic acid, quercetin, rutin, and caftaric acid had been reported [21,23], as well as saponins such as Agavoside A and Agavoside B [24]. Despite the discovery of a plethora of flavonoids and triterpenes, the absence of previously documented compounds may be attributed to the disparate methodologies employed in the extraction process. The majority of studies utilize the Soxhlet system with highly polar solvents such as ethanol or methanol, whereas our extract was obtained with ethyl acetate, an intermediate polarity solvent that facilitates the isolation of diverse bioactive compounds. This phenomenon has also been observed in another study [23], where temperature and ultrasound treatments altered the metabolite expression profile. Nevertheless, the presence of fatty acids has been documented in other agave species such as *Agave angustifolia,* where in a fraction with acetone and a methanolic subfraction enriched in fatty acids, the presence of palmitic acid, linoleic acid, and oleic acid has been reported [28]. Similarly, in an ethanolic extract of *Agave applanate*, the presence of palmitic acid, linoleic acid, and oleic acid has been reported, and a fraction with dichloromethane has been reported, as well as palmitic acid, linoleic acid, sitosterol, stigmasterol, and hecogenin [29].

In silico studies indicate that the extract may interact with α-glucosidases and DPP4; these in silico interactions lay the groundwork for focusing studies to test a likely mechanism of action of the extract. However, it is not possible to know whether the compounds will be agonists or antagonists from in silico studies. It is believed that the extract could bind and inhibit these targets due to previous reports on the biological activities of the compounds present in the extract.

The α-glucosidases such as maltase-glucoamylase and sucrase-isomaltase are responsible for breaking the α-glucoside bonds and releasing α-glucose, which is absorbed in the intestine and passes into the bloodstream. Inhibiting these enzymes delays or inhibits glucose absorption in the small intestine, thus reducing postprandial blood glucose concentrations [30]. Among the compounds present in the extract that bind to and inhibit α-glucosidases, we identify arjungenin [31,32,33], scutellarein [34,35,36], sakuranetin [37], isoimperatorin [38], ursolic acid [39,40,41], betulonic acid [42,43], rhein [44], and fucoxanthin [45], where five of these metabolites are the majority in the agave extract.

Another proposed mechanism is the inhibition of DPP4; this enzyme is responsible for the regulation of the incretin hormones glucagon-like peptide 1 (GLP1) and gastric inhibitory polypeptide (GIP) by hydrolyzing these proteins. Inhibiting DPP4 increases the half-life of GLP1, thereby stimulating insulin secretion after food intake (the so-called incretin effect), while at the same time reducing the counter-regulatory hormone, glucagon, and thus decreasing glucose production by the liver [46]. The bioactive metabolite present in the extract that has been reported as a probable DPP4 inhibitor is arjungenin; this is important since it is one of the three main major metabolites of the extract [47].

The aforementioned evidence indicates that the extract may possess inhibitory properties against DPP4 and alpha glucosidases. Consequently, an in vitro evaluation of DPP4 inhibition was conducted, as this represents a distinct mechanism compared to that observed in the majority of plants with antidiabetic activity. In vitro and in vivo assays showed that the extract inhibited DPP4, which corroborates the in silico predictions. This inhibition is probably due to triterpenes, since of the molecular docking of the thirteen bioactive compounds that interact with DPP4, seven are triterpenes and three are terpene derivatives, and it has already been reported that this family of bioactive compounds is able to inhibit DPP4 [48,49]. Although there are no studies focused on this target for other agave species, a very varied range has been reported in the IC_50_ in extracts of natural origin, ranging from 1.65 mg/mL for an ethanolic extract of *Urena lobate* [50] to 70.9 µg/mL for a methanolic extract of *Allium sativum* [51]; therefore, the extract having an IC_50_ of 861 µg/mL would be within an intermediate value.

Within this study, it was decided to include sitagliptin as a positive control in the in vivo assays; this drug is a commercial DPP4 inhibitor, which allows comparing its effectiveness against the extract, since it has the same mechanism of action. Metformin was also included as a control since it is the most widely used drug for the treatment of diabetes, which among its multiple mechanisms of action activates AMPK, generating a signaling cascade that ends in the sensitization to insulin in the receptor, allowing this hormone to carry out its function correctly, thus reducing glucose levels in peripheral blood [52]. The in vivo results showed that administration of the extract significantly reduced several parameters such as glycemia and lipid profile, showing a similar trend to that reported for sitagliptin. This was expected as they share the same mechanism of action. In addition, the extract showed lower levels of glycemia and lipids compared to metformin, since the mechanisms of these are different, and it has been reported that DPP4 inhibitors allow better control of diabetes than metformin. The combination of the extract with these drugs was also evaluated in order to assess whether the extract decreased the efficacy of these drugs or, on the contrary, improved the biochemical parameters. In this aspect, we observed that there was no great difference in the laboratory values when EAD and sitagliptin were administered together, since the therapeutic objective is the same; however, it should be noted that the therapeutic efficacy is preserved so that the extract does not seem to interfere with the effectiveness of sitagliptin. On the other hand, it is interesting that the combination of the extract with metformin produced a greater decrease in several parameters compared to when only metformin was administered, which would indicate that the use of the extract with this drug could help to better control diabetes. This is because the mechanisms of action are different—on the one hand, the extract would increase the amount of insulin in an indirect way, as well as the decrease in counter regulatory hormones, and on the other hand, metformin would improve insulin sensitivity. This type of combination of DPP4 inhibitors with metformin already exists on the market and has shown good control for diabetes, so the use of the extract with metformin could be useful for the treatment of diabetes.

Few studies have focused on diabetes and agave extracts. Among them, we found that extracts derived from *Agave lechugilla* and *Agave americana* show a reduction in triglyceride levels consistent with the findings of this study [53,54]. However, it is noteworthy that the aqueous extract of *Agave lechugilla* guishe [53] did not demonstrate a similar effect on glucose levels. These results on the decrease in triglyceride levels in the *Agave lechuguilla* guishe extract are attributed to saponins and mostly to flavonoids; however, *Agave durangensis* extract is poor in flavonoids so it could not be the family of bioactive compounds responsible for this effect. On the contrary, it is attributed to fatty acids and triterpenes, since the latter are the ones that probably inhibit DPP4. DPP4 inhibitors are known to reduce triglyceride synthesis in the liver and its accumulation, in addition to lowering total cholesterol, so this could be the most likely cause of the decrease in lipids after the administration of the extract [55,56].

On the other hand, the methanolic extract of leaves of *Agave americana* var. Marginata reduces glucose levels [54] to levels similar to those reported in this study; however, it is important to note that for *Agave americana*, the decrease was reported at a higher dose than the one we administered for *Agave durangensis*. For the case of *Agave americana,* the effect on glucose is attributed to the polyphenols and flavonoids present in the extract since they possess 51.44 ± 16 mg GAE/g of phenols and 24.95 ± 1.41 mg QE/g of flavonoids, while in our extract, these bioactive compounds were found in very small amounts of only 0.019 ± 0.000 mg GAE/g of phenols and 0.004 ± 0.000 mg QE/g of extract of flavonoids, so the effect could not be attributable to this family of bioactive compounds. We propose that the effect of the *Agave durangensis* extract is mainly due to triterpenes, since they are the second most important family of the extract, with 294.30 ± 0.88 mg GAE/g representing almost 30% of the total composition, and as previously discussed, these could be responsible for the inhibition of DPP4.

## 4. Materials and Methods

### 4.1. Collection Plant and Preparation of the Extract

*Agave durangensis* Gentry leaves were collected in the municipality of Nombre de Dios, Durango, Mexico. A specimen was deposited at the National Herbarium of Mexico (MEXU), National Autonomous University of Mexico (http://datosabiertos.unam.mx/IBUNAM:MEXU:1307193, Lat: 23. 70606° Log: −104.21086°, Gerardo Adolfo Salazar Chavez, gasc@ib.unam.mx, accessed on 15 January 2025) under the folium 1307193.

The leaves were subjected to a process of cleaning and drying in a solar dryer with no direct contact with the sun’s rays, subsequent to the crushing of the material.

The *Agave durangensis* Gentry extract (EAD) was prepared by macerating the plant material in ethyl acetate (7 L per kg of plant material) for one week at room temperature three times. The extract was then filtered and concentrated in a Büchi R-215 rotary evaporator until it reached a dry state. This process was coupled to a Büchi V-700 vacuum pump and an F-105 recirculator.

### 4.2. Phytochemical Characterization of Agave durangensis Extract

#### 4.2.1. Triterpene Quantification

For the determination of triterpenes, 10 μL of extract (1 mg/mL) was placed in each well of the microplate, followed by the addition of 15 μL of glacial acid–vanillin solution (5% *v*/*v*) and 50 μL of perchloric acid solution. The reaction mixture was incubated for 45 min at 60 °C. After cooling to room temperature, 225 μL of glacial acetic acid was added. The control contained 100% methanol in place of the sample. The absorbance was measured at 548 nm, using a microplate reader. A standard curve constructed with six concentrations of ursolic acid (UA) was used to estimate the total triterpent content in the extract. The experiment was performed in triplicate in three different determinations, and the result was expressed as mg equivalents of ursolic acid per g dry extract (mg UAE/g extract) ± standard error of the mean (SEM) [57].

#### 4.2.2. Phenol Quantification

For quantification of total phenols, 100 μL of extract (10 mg/mL) was mixed with 500 μL of Folin–Ciocalteu reagent (1:10 in water). After 4 min, 800 μL of a saturated sodium carbonate solution was added. The mixture was stirred and allowed to stand for 2 h, and the absorbance was measured at 725 nm in a spectrometer. A standard curve constructed with six concentrations of gallic acid (GA) was used to estimate the total phenolic contents in the extracts. The experiment was performed in triplicate, and the result was expressed in mg equivalents of gallic acid per g of dry extract (mg GAE/g of extract) ± SEM [58].

#### 4.2.3. Flavonoid Quantification

For flavonoid determination, 500 μL of extract (10 mg/mL) was mixed with 500 μL of 2% AlCl_3_; the mixtures were shaken and, after 1 h in the dark, the absorbance was read at 420 nm in a spectrometer. A standard curve constructed with six concentrations of quercetin (Q) was used to estimate the flavonoid contents in the extracts. The experiment was performed in triplicate, and the result was expressed in mg equivalents of quercetin per g of dry extract (mg QE/g of extract) ± SEM [59].

#### 4.2.4. Condensed Tannin Quantification

For tannin estimation, aliquots of 125 μL of extract (50 mg/mL) were mixed with 750 μL of 4% vanillin solution (dissolved in absolute methanol) and 375 μL of concentrated HCl. After 20 min, absorbance was recorded at 500 nm on a spectrophotometer. The total condensed tannins concentration in the extract was estimated from a calibration curve constructed with six concentrations of epicatechin (E) as standard. The experiment was performed in triplicate, and the result was expressed as mg equivalents of epicatechin per g of dry extract (mg EE/g of extract) ± SEM [60].

#### 4.2.5. UPLC-MS Analysis

The mass spectrometry analysis was conducted on an Acquity UPLC I-class instrument with a diode array detector coupled to a mass spectrometer with an ESI ionization source and a Waters brand VION IMS time of flight.

The analysis was carried out under the following conditions: the sample was diluted in mass grade methanol to a concentration of 1 mg/mL. The column used was BEH C18 2.1 × 100 mm, 1.7 μm. The mobile phases used were acetonitrile (Phase A) and water (Phase B), both acidified with 0.1% formic acid. The gradient used is described in Table 6 with a flow rate of 0.4 mL/min, column temperature of 35 °C, sample temperature of 10 °C, and injection volume of 2 μL.

An absorbance scan was carried out from 210 to 600 nm, with specific channels: 214, 280, 320, 360 (and 520 nm in positive mode). Ionization was carried out in positive and negative modes. The mode of analysis was MSE, in which the low collision energy was 6 eV, with a ramp from 15 to 45 eV at high energy. The range of masses was considered from 50 to 1800 *m*/*z*. The capillary voltage used was 2 and 3.5 kV for negative and positive, respectively; source temperature 120 °C; desolvation temperature 450 °C; argon as collision gas; desolvation with flows of 50 L/h and 800 L/h, respectively; and a cone voltage of 40 V. Leucine enkephalin was used at a concentration of 200 pg/μL as a reference for mass correction with a flow of 10 μL/min.

The Unifi 1.9 SR 4 Software was employed for the purpose of data analysis, utilizing libraries from the Food Analysis Specialized Laboratory (LUCAL), the University of Mississippi Botanical Library, and the University of Ottawa Phytochemical Library. The target coincidence tolerance was established at 5 ppm. In order to identify the fragments, a comparison was made with the fragmentation patterns reported in PubChem, FooDB version 1.0 and MDB version 5.0.

### 4.3. In Silico Study

#### 4.3.1. Obtaining and Preparation of Proteins and Ligands

Human proteins related to the main targets of the main groups of oral drugs for the treatment of diabetes mellitus were used as described in Table 7, being obtained from the Research Collaborator for Structural Bioinformatics Protein Data Bank (RCSB PDB) (https://www.rcsb.org, accessed on 2 November 2022). Prior to the molecular coupling, the protein files were prepared in PyMOL, eliminating the ligands and water molecules, and the file was finished in AutoDockTools (version 1.5.7), where the polar hydrogens were added in those atoms capable of establishing bridge-type interactions of H (O and N atoms) and the Kollman charges considering the complete protein to finally be saved in the format .pdbqt.

The 3D structures of the compounds identified by the UPLC-MS analysis as well as that of the controls were obtained in SDF format from the PubChem database (https://pubchem.ncbi.nlm.nih.gov, accessed on 2 November 2022) with the exception of the compounds ursolic acid 3-arabinopyranoside [61], spinogenin C6 and C7, and spinoside C3 [62], whose 3D structures were not found in the PubChem database, so they were obtained from the SMILES code, which was converted to .sdf format in the OPENBABEL program (version 2.4.0) [63]. Later, they were converted into .pdb format in PyMOL to finally be prepared within the AutoDockTools software (version 1.5.7), where the Gasteiger loads were added and the rotatable links were established, and finally, they were saved in .pdbqt format.

#### 4.3.2. Molecular Docking

Docking was run using the AutoDock Vina program (version 1.1.2) [64] to predict the affinity between compounds identified by UPLC-MS at the active site of the protein. The docking specifications are reported in Table 7.

The co-crystallized ligands of each protein were re-docked to each other using the reported coordinates on the crystal as the center for each grid. The grid box dimensions for X, Y, and Z were 40 Å × 40 Å × 40 Å with a grid point spacing of 0.375 Å. Conversely, the completeness and the number of modes were 8 and 9, respectively. The same criteria were applied for the coupling of the various targets to the compounds with the various targets.

Those compounds that had a lower delta G than the controls of each of the blanks were selected. The molecular docking results were analyzed using the Biovia Discovery Studio Visualizer software (version 20.1.0.19295).

#### 4.3.3. In Silico Pharmacokinetic Evaluation of the Investigated Compounds

Pharmacokinetic properties are important parameters to determine the effectiveness of drugs. Drugs that show good absorption, distribution, metabolism, excretion, and toxicity (ADMET) parameters are more likely to become regulatory-approved drugs. For the in silico prediction of the ADME parameters of the compounds found in the extract of *Agave durangensis* Gentry leaves, the ADMETlab 2.0 server (https://admetmesh.scbdd.com/, accessed on 2 November 2022) [65] was used, and the ProToxII server (https://tox.charite.de/protox_II, accessed on 2 November 2022) [66] was employed for the prediction of toxicity in both the SMILES codes of each compound to obtain the desired information. This included physicochemical properties, gastrointestinal absorption, blood–brain barrier penetration, Caco-2 cell permeability, substrate status, inhibition of GP protein, inhibition of some P450 family cytochromes, and a range of other parameters. These included oral LD_50_, mutagenicity, carcinogenicity, hepatotoxicity, and cytotoxicity.

### 4.4. In Vitro Study

#### Dipeptidyl Peptidase-4 Inhibition Assay

DPP-IV inhibitory activity was determined using the DPP-IV inhibitor detection kit MAK203 (Sigma Aldrich, Missouri, St Louis, MO, USA). The reaction mixture (100 µL), containing 50 µL of enzyme solution, 25 μL EAD, buffer or sitagliptin (at different concentrations), and 25 µL of substrate, was incubated at 37 °C for 30 min, and fluorescence was recorded every minute (FLU, λex = 360/λem = 460 nm). The result was expressed as IC_50_ value, this being the concentration required to inhibit 50% of DPP-IV activity.

### 4.5. In Vivo Study

#### 4.5.1. Evaluation of the Hypoglycemic Effect of EAD

Adult male Wistar rats, aged eight weeks and with a mean body weight of 250 ± 50 g, were sourced from the FES Iztacala UNAM Bioterio. They were maintained under standard conditions at 22–23 °C and with light–dark periods of 12 h/12 h. The animals were fed standard diet (5001 Labdiet@ Rodent Laboratory Chow, Indiana, Richmond, VA, USA) and water. The Bioethics Committee of the National School of Medicine and Homeopathy of the National Polytechnic Institute approved this research project (CBE/001/2022), which complies with International Standards and Policies.

A single intraperitoneal (i.p.) injection of streptozotocin (STZ, Sigma Aldrich, Missouri, St Louis, MO, USA) prepared with sodium citrate buffer at pH = 4.5 at a dose of 50 mg/kg was administered to induce hyperglycemia in rats. Forty-eight hours following the injection, blood glucose levels were assessed using a glucometer (Accucheck Active Roche^®^). Animals with blood glucose levels exceeding 150 mg/dL were classified as diabetic [67]. To assess the antihyperglycemic effect, all treatments were administered via intragastric cannula. The animals were classified into seven groups, each comprising six animals, as follows: healthy; diabetic; sitagliptin (diabetic treated with sitagliptin at a dose of 10 mg/kg); metformin (diabetic treated with metformin at a dose of 100 mg/kg); EAD (diabetic treated with EAD at a dose of 300 mg/kg); EAD + sitagliptin (diabetic treated with the combination of EAD at a dose of 300 mg/kg with sitagliptin at a dose of 10 mg/kg); and EAD + metformin (diabetic treated with the combination of EAD at a dose of 300 mg/kg with metformin at a dose of 100 mg/kg). All treatments were prepared in dimethyl sulfoxide (DMSO, Sigma-Aldrich D4540, Missouri, St Louis, MO, USA) in water USP (1:3) and were administered on a daily basis for 30 days. After the treatment period, the animals were sacrificed according to the American Veterinary Medical Association (AVMA) guidelines for the euthanasia of animals [68]. Additionally, blood samples were collected for biochemical testing, and small tissue samples of the pancreas, liver, kidney, and intestine were obtained for histopathological examination.

#### 4.5.2. Biochemical Study

At the end of the treatments, the animals were sacrificed by cardiac puncture after being anesthetized with ketamine/xylazine (40:5 mg/kg); the blood was collected with and without anticoagulant. Serum was obtained by centrifugation at 570× *g* for 10 min at 4 °C, and it was processed in the AutoKem-II system (KontroLab, Rome, Italy). Biochemical parameters included serum glucose levels; liver enzymes such as aspartate amine transferase and alanine amine transferase; lipid profile including triglycerides, cholesterol total, high-density lipoproteins (HDL), low-density lipoproteins (LDL), and very-low-density lipoprotein; and kidney function based on urea and creatinine levels, as well as glycated hemoglobin. Insulin levels were calculated with the use of the kid EZRMI-13K (MERK-MILLIPORE).

#### 4.5.3. Dipeptidyl Peptidase-4 Activity Assay

The ab204722 kit was used to determine DPP4 activity in serum. The kit recommendations were followed where plasma samples were diluted directly in DPP-4 assay buffer. Samples were adjusted to a final volume of 50 μL in a 96-well plate using DPP-4 assay buffer, and sitagliptin was used as a positive control. A total of 10 μL of buffer or control was added to each well, and the mixtures were incubated at 37 °C for 10 min. Finally, the reaction mixture (40 μL) was added to each well, and the mixtures were incubated at 37 °C for 30 min. Fluorescence measurement (λex/λem = 360/460 nm) was performed in kinetic mode for 30 min. The results were analyzed according to the manufacturer and expressed as percentage activity.

#### 4.5.4. Histological Studies

Internal organs, including the pancreas, kidneys, liver, and intestines, were processed for histopathological examination and stained with hematoxylin and eosin (H&E).

### 4.6. Statistical Analysis

The experimental data for antidiabetic activity were subjected to one-way analysis of variance, followed by Dunnett’s test. * *p* values ≤ 0.05, ** *p* ≤ 0.005, *** *p* ≤ 0.001 were considered statically significant. We used GraphPad Prism version 5.0 (GradphPad Software, Inc.).

## 5. Conclusions

This study discovered the bioactive compounds present in the leaves of *Agave durangensis*, where linolenic acid is the major metabolite, followed by spinogenin C7 and arjungenin, two triterpenes, whose family represents 30% of the total composition of compounds in the extract. It was demonstrated that the extract at a dose of 300 mg/kg decreased the levels of glucose and glycated hemoglobin as commercial drugs do, besides the fact that the extract has a beneficial effect on lipid levels, allowing the decrease in parameters such as VLDL cholesterol and triglycerides in a similar way as reported in other agave extracts. These effects are mainly attributed to the triterpenes and their activity on DPP4 since in silico analyses show that this family of bioactive compounds is capable of interacting with this therapeutic target and in vitro and in vivo assays show that the extract inhibits this enzyme; therefore, one of the probable mechanisms of action of the extract is the inhibition of DPP4. Finally, co-administration of the extract with diabetic drugs does not seem to decrease efficacy or cause side effects; on the contrary, when administered with metformin, a greater decrease in glucose levels was observed than when metformin alone was administered. Based on this evidence, *Agave durangensis* extract could be used for the development of phytomedicines or as a source of molecules for the treatment of diabetes, but studies are needed to demonstrate its safety when administered.

## Figures and Tables

**Table 1:** Characterization by ultra-performance liquid chromatography–mass spectrometry positive mode.

Adduct	Compound	Observed Mass (g/mol)	Expected Mass (g/mol)	Mass Error (ppm)	Tr (min)	Response	Fragment Ions (Positive Mode) (*m*/*z*)								
(+H)	Spinogenin C7	472.3192	472.32	0.8	19.84	73,110	455.3159	437.30634	341.24792						
(+H)	Ursolic acid 3-arabinopyranoside	588.4022	588.4	−0.6	21.67	10,557	119.08552	105.07013	221.1335	303.30564					
(+H)	Spinoside C3	928.5034	928.5	0.3	14.7	6801	207.03239								
(+H)	Trihydroxylup-20-en-28-oic acid	488.3521	488.35	3.9	23.89	5853	335.25784	261.22124	333.24281	259.20575	173.13234	145.10116			
(+H)	Acanthosessilioside C	694.3909	694.8	−2.7	11.45	5834	661.39018								
(+H)	Spinogenin C6	474.3353	474.33	1.7	19.94	5740	89.06008	335.25978	379.28394	439.32245					
(+H)	Makisterone A	494.3223	494.7	−4.1	22.11	5700	255.23218	221.08592	165.09117						
(+H)	Arjungenin	504.3456	504.7	1	21.28	5557	333.24176	437.33973							
(+H)	Pregna-5,14-diene-3b,20a-diol	316.2409	316.24	2.2	16.21	4951	299.23659	281.22635	187.14858	173.13401	145.10141	105.07021	215.14367	225.16449	269.19088
(+H)	Belladonnine	542.3141	542.7	−0.6	12.19	3833	189.09477	101.04227	233.12259						

**Table 2:** Characterization by ultra-performance liquid chromatography–mass spectrometry negative mode.

Adduct	Compound	Observed Mass (g/mol)	Expected Mass (g/mol)	Mass Error (ppm)	Tr (min)	Response	Fragment Ions (Negative Mode) (*m*/*z*)						
(−H)	Linolenic acid	278.2248	278.4	0.8	18.35	622,876	278.22081	277.21751	259.20662				
(−H)	Arjungenin	504.3443	504.7	−1.6	21.25	55,882	277.21753	293.21236	419.3143	471.30954	235.16928	471.30954	443.3136
(−H)	D-alpha-Tocotrienol	424.3344	424.7	0.5	21.7	38,951	205.15989	145.02954					
(−H)	Vachanic acid	252.1726	252.35	0.3	18.36	23,819	221.15407	220.14654	149.09725	171.10206			
(−H)	10-Gingerol	350.244	350.5	−5	20.86	21,577	149.09705	236.1041	275.20213				
(−H)	Scutellarein	286.0477	286.24	0	8.95	19,940	285.0406	255.02907	283.02385				
(−H)	Sakuranetin	286.0843	286.28	0.8	9.44	19,333	179.03465	285.07816	177.01968				
(−H)	Isoimperatorin	270.0893	270.28	0.2	12.31	15,674	269.08187	254.05786					
(−H)	Ursolic acid	456.3604	456.7	0.2	23.92	15,275	455.35314	456.35654	457.36112	453.33631			
(−H)	Betulonic acid	454.3449	454.7	0.4	23.19	11,434	277.2178	339.20073	438.31414				
(−H)	Petroselinic acid methyl ester	296.2718	296.5	1	21.43	8831	149.09754						
(−H)	Ricinoleic acid	298.2509	298.5	0.3	16.15	7396	279.23256	297.24312	221.15402				
(−H)	Rhein	284.0322	284.22	0.2	8.96	7153	183.01199	283.02385	211.03993				
(−H)	Caffeic acid	180.04226	180.16	1.5	9.44	5464	179.03465	135.0449					
(−H)	Lignoceric acid methyl ester	382.3808	382.7	−0.9	24.77	4934	367.35847	339.32682	253.21758	225.22208			
(−H)	Caffeic acid 3-glucoside	342.0955	342.3	1.3	3.78	4443	179.03453	135.04567					
(−H)	Hoodistanaloside A	914.4909	915.1	3.7	7.24	4339	623.41572	867.47919	837.43031				
(−H)	Cnicine	378.1693	378.4	3.7	15.72	4247	309.17442	119.05023					
(−H)	Fucoxanthin	658.4262	658.9	4.4	21.9	4163	605.40462	537.3761	486.28544	275.20176			
(−H)	Acankoreoside B	958.5157	959.1	2	7.37	3903	881.45779	911.50138					
(−H)	Linoelaidic acid methyl ester	294.2559	294.5	0	20.18	3894	277.21713	279.23301	149.09723				
(−H)	Vachanic acid methyl ester	266.1881	266.38	−0.3	18.35	1872	221.15407	233.11473	149.09725	205.15939	171.10206		
(−H)	Spinogenin C6	474.3342	474.33	−0.6	23.17	1830	277.2178	339.20073	391.28602	438.31414			
(−H)	Isoodoratol	474.372	474.7	2.3	22.41	1674	279.23321	367.29661	293.21262	227.20168			
(−H)	Melianone	470.3391	470.7	−1.2	20.93	1503	293.21165	353.20613	275.20213	185.11715			
(−H)	Acanthosessilioside D	648.3885	648.8	1.8	20.47	1498	577.3741	503.33659	485.32631	309.20769	291.1963		
(−H)	11,13-Dihydroparthenolide	250.1566	250.33	−1	16.79	1490	149.09753	205.15907					
(−H)	Bakkenolide A	234.1619	234.33	−0.4	20.79	1292	221.15462	205.16					

**Table 3:** Free energy of binding interaction (kcal/mol) of bioactive compounds from *Agave durangensis* Gentry extract with target proteins.

Compound	AMPK	SUR1	α-Glucosidases				PPARγ	DPP4	SGLT2	GLP1-R
			C-Terminal Subunit of Maltase-Glucoamylase	N-Terminal Subunit of Maltase-Glucoamylase	Sucrase-Isomaltase	Pancreatic α-Amylase				
Linolenic acid	−5.4	−6.1	−4.9	−4.7	−5.1	−4.9	−4.8	−5.4	−5.2	−3.9
Spinogenin C7	−7.3	−8.5	**−8.8**	**−7.8**	−6.5	**−9.1**	−8.1	−7.9	−8	−8.4
Arjungenin	−7.5	−9.2	**−8.5**	**−6.9**	**−7**	**−9**	−8	**−8.6**	−7.9	−8.2
D-α-Tocotrienol	−5.4	−8.8	**−8.2**	**−8**	**−7.1**	**−8.5**	−8.4	−8	**−10.8**	−7.1
Vachanic acid	−6.1	−7.2	−6.8	−6.6	**−6.9**	−7.7	−7	−7.4	−7.2	−9
10-Gingerol	−7	−6.7	−6.9	−5.8	−6.4	−6.3	−5.8	−5.9	−8.3	−8.3
Scutellarein	−9.4	−8.2	**−9**	**−7.3**	**−7.7**	**−8.4**	−7.4	−7.8	−8.8	−9.1
Sakuranetin	−10.6	−8.9	**−9.3**	**−8.3**	**−8.5**	**−9.7**	**−8.7**	**−8.6**	−10.5	−9.6
Isoimperatorin	−8	−7.5	**−7.8**	**−7.1**	**−7.5**	**−7.8**	−7.1	−7.7	−9.6	−6.8
Ursolic acid	−7.7	−9.4	**−8.1**	**−7.5**	**−7**	**−9.3**	−7.9	**−8.7**	−9.5	−8.9
Betulonic acid	−7.8	−9.1	**−8.8**	**−7**	**−7**	**−9.1**	−8	−8.3	−8.2	−8.2
Ursolic acid 3-arabinopyranoside	−7.5	**−9.8**	**−9.2**	**−7.6**	**−8.1**	**−9**	**−8.8**	**−8.6**	−9.2	−8.9
Petroselinic acid methyl ester	−5.3	−5.3	−5.7	−4.4	−4.1	−3.8	−3.8	−4.6	−6.7	−5.5
Ricinoleic acid	−5.6	−5.8	−5.7	−5.4	−5.5	−5.1	−6.2	−5.2	−6.7	−6.8
Rhein	−9.3	−8.6	**−9**	**−6.9**	**−7.4**	**−8.7**	−7.7	**−8.5**	−9	−9.6
Spinoside C3	−7.3	**−10.7**	**−9.2**	**−7.6**	**−8.6**	**−9**	**−9.4**	**−9.4**	−9.6	−9
Trihydroxylup-20-en-28-oic acid	−7.4	−8.6	**−8.6**	**−7.1**	**−7.1**	**−8.7**	−8.2	−8.4	−8.1	−7.6
Acanthosessilioside C	−7.1	−8.6	**−7.9**	**−7.4**	**−7.2**	**−8.5**	−8	−8.4	−8.3	−8.1
Makisterone A	−7.7	−8.5	**−8.4**	**−6.7**	−6.4	**−8.5**	**−8.9**	−8.2	−9.1	−9
Caffeic acid	−6.7	−6.5	−6.9	−6.2	−6.3	−6.5	−6.6	−6.8	−7.6	−6.7
Pregna-5,14-diene-3b,20a-diol	−7.2	−8.2	**−7.6**	**−7**	−6.5	**−8.7**	−7.1	−8.1	−8	−10.3
Lignoceric acid methyl ester	−3.3	−5.5	−5	−4.7	−4.6	−4.4	−5.1	−5.2	−6.7	−7.1
Caffeic acid 3-glucoside	−7.9	−7.5	**−8.3**	**−7.3**	−6.6	−7.6	−6.7	−8.4	−9	−7.6
Hoodistanaloside A	−7.6	**−9.6**	**−10.1**	**−7.8**	**−7.5**	**−9.9**	**−8.9**	**−9.8**	−8.7	−8.2
Cnicine	−6.4	−8.4	−7.4	**−7**	−6.6	−7.7	−6.8	−8.4	−7.3	−7.2
Fucoxanthin	−7.9	−2.3	**−8.3**	**−7.2**	**−7.6**	**−8.8**	−7.5	**−9.2**	**−11**	−8.6
Acankoreoside B	−9.4	**−10.3**	**−10.2**	**−8.5**	**−7.7**	**−11.1**	**−10.1**	**−11.5**	−10.2	−10.5
Linoelaidic acid methyl ester	−3.8	−5.8	−5.2	−5.1	−5	−5.4	−5.5	−5.1	−6.2	−7.1
Belladonnine	−7.7	**−10.1**	**−10.5**	**−8.4**	**−8.8**	**−8.5**	**−9**	**−9.6**	−8.9	−8.3
Vachanic acid methyl ester	−5.7	−6.9	−6.9	−6.2	−6.4	−7.7	−6.9	−6.8	−7.9	−9
Spinogenin C6	−6.9	−8	**−8.2**	**−7.5**	**−7.7**	**−10.4**	−7.6	−8.4	−8.5	−8.4
Isoodoratol	−7.4	−9.1	**−9.3**	**−7.7**	**−7**	**−9.6**	**−8.8**	**−8.9**	−9.6	−8.6
Melianone	−8.3	**−9.6**	**−8.9**	**−8.2**	**−8.1**	**−10.4**	−8.5	**−9.2**	−9.6	−8.7
Acanthosessilioside D	−8	−8.7	**−8.5**	**−8.2**	**−7.1**	**−9.3**	**−8.7**	**−9.2**	−8.9	−8.9
11β,13-Dihydroparthenolide	−8.5	−7.3	−7.2	−6.1	−5.8	−7.4	−6.9	−7.8	−7.6	−9
Bakkenolide A	−7	−7.6	−6.5	−5.5	−5.9	−7.6	−6.8	−8.3	−7.6	−7.1
Metformin	**−4.9**									
CG7	**−10.9**									
Glibenclamide		**−9.6**								
Acarbose			**−7.6**	**−6.7**	**−6.7**	**−7.8**				
Pioglitazone							**−8.7**			
Sitagliptin								**−8.5**		
Empagliflozin									**−10.7**	
Danuglipron										**−13.1**

**Table 4:** Biochemical profile from rats after 30 doses of *Agave durangensis* extract (EAD) and combinations.

	Healthy	Diabetic	Sitagliptin	Metformin	EAD	EAD + Sitagliptin	EAD + Metformin
Glucose (mg/dL)	104.30 ± 5.54 ***	407.00 ± 60.21	171.70 ± 25.83 **	195.00 ± 50.32 **	176.00 ± 52.58 **	157.30 ± 8.34 **	154.70 ± 11.70 **
HbA1c (%)	4.80 ± 0.28 **	9.68 ± 1.14	6.27 ± 0.82 *	7.45 ± 1.01	4.62 ± 0.54 ***	6.55 ± 0.23 *	6.43 ± 0.32
Insulin (ng/mL)	0.18 ± 0.02	0.10 ± 0.02	0.05 ± 0.01	0.06 ± 0.02	0.10 ± 0.02	0.14 ± 0.02	0.08 ± 0.02
Urea (mg/dL)	90.67 ± 6.64 **	265.00 ± 36.67	182.00 ± 19.05	225.00 ± 35.04	236.30 ± 38.34	181.00 ± 9.31	200.30 ± 41.06
Creatinine (mg/dL)	1.00 ± 0.05	1.97 ± 0.51	1.26 ± 0.06	1.26 ± 0.08	2.02 ± 0.53	1.15 ± 0.08	1.13 ± 0.18
Total cholesterol (mg/dL)	55.00 ± 6.50	77.25 ± 7.60	39.33 ± 4.41 **	48.33 ± 7.31 *	45.67 ± 4.25 *	42.50 ± 9.02 **	51.00 ± 5.68
HDL (mg/dL)	14.00 ± 1.52	19.25 ± 1.93	10.00 ± 1.15 **	12.33 ± 1.76	11.67 ± 1.20 *	10.50 ± 2.25 **	13.00 ± 1.52
LDL (mg/dL)	26.33 ± 2.60	36.00 ± 1.68	21.67 ± 2.02	26.00 ± 5.85	25.00 ± 3.78	24.25 ± 5.89	29.00 ± 4.72
VLDL (mg/dL)	14.67 ± 3.18	22.00 ± 4.02	7.66 ± 2.18 **	10.00 ± 2.88 *	8.75 ± 2.05 **	7.75 ± 1.31 **	9.00 ± 2.08 *
Triglycerides (mg/dL)	72.33 ± 15.59	109.30 ± 20.34	38.67 ± 10.37 **	49.67 ± 13.59 *	44.50 ± 10.52 **	39.00 ± 7.29 **	44.67 ± 9.61 *
Total bilirubin (mg/dL)	0.16 ± 0.03 **	0.35 ± 0.01	0.23 ± 0.03	0.29 ± 0.03	0.29 ± 0.04	0.41 ± 0.04	0.32 ± 0.03
Direct bilirubin (mg/dL)	0.08 ± 0.02	0.11 ± 0.03	0.15 ± 0.05	0.20 ± 0.04	0.11 ± 0.01	0.17 ± 0.01	0.17 ± 0.01
Indirect bilirubin (mg/dL)	0.07 ± 0.01 *	0.23 ± 0.03	0.08 ± 0.02 *	0.09 ± 0.00 *	0.18 ± 0.04	0.23 ± 0.03	0.15 ± 0.03
AST (U/L)	244.00 ± 8.08	812.50 ± 217.50	686.70 ± 159.20	649.30 ± 155.50	582.50 ± 122.80	400.00 ± 169.40	475.00 ± 95.00
ALT (U/L)	280.70 ± 11.10	463.00 ± 91.14	317.30 ± 23.25	378.70 ± 73.54	380.50 ± 63.54	185.00 ± 35.71 *	275.00 ± 15.00

Results are expressed as mean ± SEM (*n* = 6). * *p* < 0.05; ** *p* < 0.01; *** *p* < 0.001 compared to the diabetic group.

**Table 5:** Percentage of DPP4 activity in serum.

Healthy	Diabetic	Sitagliptin	Metformin	EAD	EAD + Sitagliptin	EAD + Metformin
100.00%	110.90 ± 1.44%	7.13 ± 1.70% ***	128.40 ± 8.68%	74.72 ± 3.58% ***	4.54 ± 1.97% ***	94.86 ± 7.89%

Results are expressed as mean ± SEM (*n* = 4). *** *p* < 0.001 compared to the diabetic group.

**Table 6:** Gradient used for UPLC-MS analysis.

Time (Minutes)	Phase A	Phase B
0	95	5
2	95	5
22	5	95
25	5	95
27	95	5
30	95	5

**Table 7:** Specifications of targets, controls, and characteristics of molecular docking.

Drug Family	Enzyme Target	PDB	Control	Coordinates of the Grid Box			Size of the Grid Box
				X	Y	Z	
Biguanides	AMPK	6B1U	CG7	−48.97	27.95	−67.13	40 Å × 40 Å × 40 Å
Sulfonylureas	SUR1	7S5V	Glibenclamide	198	296	232	40 Å × 40 Å × 40 Å
α-Glucosidase inhibitor	Maltase-glucoamylase	3TOP	Acarbose	−30.61	35.65	26.44	40 Å × 40 Å × 40 Å
		2QMJ		−20.8	−6.58	−5.07	40 Å × 40 Å × 40 Å
	Sucrase-isomaltase	3LPP		39.89	58.74	78.87	40 Å × 40 Å × 40 Å
	Pancreatic α-amylase	4W93		−9.63	4.34	−23.1	40 Å × 40 Å × 40 Å
Thiazolidinedione	PPARγ	5Y2O	Pioglitazone	−48.34	−2.16	78.2	40 Å × 40 Å × 40 Å
DPP-4 inhibitor	DPP4	1X70	Sitagliptin	41.18	51.04	35.62	40 Å × 40 Å × 40 Å
SGLT2 inhibitor	SGLT2	7VSI	Empagliflozin	38.3	50.24	46.38	40 Å × 40 Å × 40 Å
GLP-1R agonists	GLP-1 receptor	6X1A	Danuglipron	131.34	116.77	155.03	40 Å × 40 Å × 40 Å

## Data Availability

Data are contained within the article.

## Supplementary Materials

The following supporting information can be downloaded at https://www.mdpi.com/article/10.3390/plants14060894/s1. Supplementary Tables, Supplementary Table S1: Summary of binding affinity and types of molecular interactions between metabolites from *Agave durangensis* Gentry extract with proteins. Supplementary Table S2. Physicochemical and in silico ADMET properties from *Agave durangensis* Gentry extract.

## Abbreviations

The following abbreviations are used in this manuscript:

DM2	Diabetes mellitus type 2
DPP4	Dipeptidyl peptidase 4
SGLT2	Sodium-glucose cotransporter-2
GLP-1	Glucagon-like peptide-1
GIP	Gastric inhibitory polypeptide
EAD	*Agave durangensis* Gentry extract
UPLC-MS	Ultra-performance liquid chromatography–mass spectrometry
AMPK	AMP-activated protein kinase
SUR1	Sulfonylurea receptor −1
PPARγ	Peroxisome-proliferator-activated receptor gamma
STZ	Streptozotocin
AST	Aspartate amine transferase
ALT	Alanine amine transferase
HDL	High-density lipoproteins
LDL	Low-density lipoproteins
VLDL	Very-low-density lipoprotein
HbA1c	Glycated hemoglobin
H&E	Hematoxylin and eosin
